# Lysosomal P-gp-MDR1 Confers Drug Resistance of Brentuximab Vedotin and Its Cytotoxic Payload Monomethyl Auristatin E in Tumor Cells

**DOI:** 10.3389/fphar.2019.00749

**Published:** 2019-07-17

**Authors:** Peggy Liu-Kreyche, Hong Shen, Anthony M. Marino, Ramaswamy A. Iyer, W. Griffith Humphreys, Yurong Lai

**Affiliations:** Pharmaceutical Candidate Optimization, Bristol-Myers Squibb Company, Lawrenceville, NJ, United States

**Keywords:** antibody drug conjugates, P-glycoprotein (P-gp-MDR1), multidrug resistance, cytotoxicity, monomethyl auristatin E

## Abstract

Antibody-drug conjugates (ADCs) are composed of an antibody linked to cytotoxic anticancer payloads. ADCs recognize tumor-specific cell surface antigens and are internalized into lysosomes where proteolytic enzymes release the cytotoxic payloads. Efflux transporters on plasma membrane that play a significant role on multi-drug resistance in chemotherapy can be internalized on lysosomal membrane and sequester the cytotoxic payloads. In the present study, ATP binding cassette (ABC) efflux transporters including breast cancer resistance protein (BCRP), P-glycoprotein (P-gp-MDR1), multidrug resistance protein (MRP) 2, MRP3 and MRP4 in lysosomal, and plasma membrane of tumor cells were quantified by targeted quantitative proteomics. The cytotoxicity of brentuximab vedotin and its cytotoxic payload monomethyl auristatin E (MMAE) to the tumor cell lines in the presence and absence of elacridar (P-gp-MDR1 inhibitor) or chloroquine (lysosomotropic agent) were evaluated. MMAE is a substrate for P-gp-MDR1, as the apparent efflux ratio in MDR1 transfected MDCK cell monolayers was 44.5, and elacridar abolished the MMAE efflux. Cell lines that highly express P-gp-MDR1 show higher EC_50_s toward the cell killing effects of MMAE. Co-incubation with chloroquine or elacridar resulted in left shift of MMAE EC_50_ by 2.9–16-fold and 4.2–22-fold, respectively. Similarly co-incubation with chloroquine or elacridar or in combination of chloroquine and elacridar increased cytotoxic effects of brentuximab vedotin by 2.8- to 21.4-fold on KM-H2 cells that express a specific tumor antigen CD30 and P-gp-MDR1. These findings demonstrate important roles of P-gp-MDR1 on cytotoxic effects of brentuximab vedotin and its payload MMAE. Collectively, ABC transporter-mediated drug extrusion and/or sequestration needs to be early assessed for selection of optimal payloads and linkers when developing ADCs.

## Introduction

Over the past few decades, many cytotoxic small-molecule drugs have been developed for cancer chemotherapy. Despite vast progress in the field, the cytotoxicity toward both normal and malignant cells has limited their clinical utility. In addition, malignant cells develop cross-resistance to other chemotherapy cytotoxic drugs after initial exposure to a cytotoxic drug. Multidrug resistance (MDR), a phenomenon manifested as a cross-resistance to a wide variety of structurally and pharmacologically diversified anticancer drugs, has become a major issue in cancer therapy. Several mechanisms can account for the acquired MDR. Of those, drug-induced expression of ATP binding cassette (ABC) superfamily of transporters has been proven to be the major mechanism of MDR (Zhou et al., 2008; Januchowski et al., 2014). These efflux transporters can interact with many kinds of anticancer agents and extrude the cytotoxic drugs out of tumor cells to minimize the intracellular exposure (Conseil et al., 2005).

To overcome the MDR of chemotherapy, recent advances in cancer research shift the focus from traditional chemotherapy to targeted cancer therapy (Peters and Brown, 2015). Due to genomic mutations in oncogenes and/or tumor suppressors, many tumor associated antigens are found to be overexpressed in tumor cells. These proteins provide ideal targets for developing monoclonal antibody (mAb)-based therapies. mAbs specifically bound to tumor associated antigens that are highly expressed in tumor cells, as opposed to normal cells, exert their therapeutic effects through either abrogating tumor cell signalling, resulting in apoptosis, or by modulating immune cell function (Green et al., 2000). However, insufficient cytotoxic activities have been the key drawback of the mAb therapies (Reichert, 2001). As a result, further efforts in utilizing the targeting nature of mAbs to deliver a chemically or genetically conjugated toxic molecule to enhance the therapeutic efficacy have been explored (Cheong et al., 2011; Gibiansky and Gibiansky, 2014). The cytotoxic small molecules linked mAbs through chemical linkers give rise to an entirely new class of anti-cancer drugs known as antibody-drug conjugates (ADCs) (Deckert et al., 2013; Gibiansky and Gibiansky, 2014). ADCs allow for the discrimination between normal and cancer cells through the selective bindings of mAbs. As a result, an ADC drug can be designed to specifically deliver a cytotoxic reagent (also known as the “payload”) to cancer cells through targeting surface tumor antigens. Once mAbs bind to surface antigens, ADCs are internalized into endosomes and lysosomes, where further processing releases of the toxic payloads. Unfortunately, regardless of initial encouraging clinical results, this class of drugs encounters a number of challenges including inherent and acquired drug resistance (Loganzo et al., 2015). One of the hypotheses for drug resistances to ADCs is due to the efflux transporters localized on the lysosomal membranes that can transport their substrates from the cytoplasm back into the lysosomes to sequester the payloads, thereby preventing it from reaching the intended targets. Since ADC payloads are known to interact with ABC efflux transporters, these transporters expressed on the lysosomal membrane could be an additional mechanism for MDR of an ADC. The mechanism was one of the hypotheses for Mylotarg^®^ resistance in the clinic, which resulted in its withdrawal from the market due to the less optimal efficacy (Przepiorka et al., 2013). Overexpression of efflux transporter proteins in tumor cells was considered to attribute to the acquired drug resistance (Loganzo et al., 2015). As almost all the next-generation cytotoxic compounds, including monomethyl auristatin E (MMAE) and maytansine, are substrates for efflux transporters such as P-glycoprotein (Kovtun et al., 2010), it is now increasingly clear that resistance to an ADC likely follows the conventional path of small molecule anticancer agents (Shefet-Carasso and Benhar, 2015).

In the present investigation, expressions of efflux transporters in various tumor cells are determined and the cell-killing effects of brentuximab vedotin and MMAE are evaluated through modulating P-gp-MDR1 activities and lysosomal functions. The findings demonstrated that P-gp-MDR1 expressed on the plasma and/or lysosomal membrane plays a significant role in MDR of MMAE and its ADC drug, brentuximab vedotin.

## Materials and Methods

### Chemicals

ProteoExtract^®^ Native Membrane Protein Extraction Kit and human serum albumin (HSA) were purchased from Calbiochem (San Diego, CA). XTT Viability Kit was purchased from Roche Applied Science (Mannheim, German Roche Applied Science, Mannheim, Germany). The Bicinchoninic Acid (BCA) Protein Assay Kit was purchased from Pierce (Rockford, IL). Modified trypsin was purchased from Promega (Madison, WI). Ammonium bicarbonate, dithiothreitol, iodoacetamide, and formic acid (FA) were obtained from Sigma Aldrich (St. Louis, MO). Liquid chromatography–mass spectrometry (LC-MS) grade acetonitrile (ACN) and water were from Burdick & Jackson (Muskegon, MI). Brentuximab vedotin was obtained from Biologics Discovery California (BDC) of Bristol-Myers Squibb (Redwood City, CA). MMAE and elacridar were purchased from MedKoo Biosciences, Inc. (Chapel Hill, NC). Chloroquine phosphate was purchased from USP (Rockville, MD). Fetal bovine serum (FBS), 4-(2-hydroxyethyl)-1-piperazineethanesulfonic acid (HEPES) buffer, phosphate-buffered saline (PBS) buffer, and penicillin-streptomycin were obtained from Corning cellgro (Manassas, VA). RPMI 1640 medium and trypsin ethylenediaminetetraacetic acid (EDTA) were purchased from Gibco (Grand Island, NY). The peptide standards and their respective stable isotope-labeled internal standards for the five efflux transporters (BCRP, MDR1, MRP2, MRP3, and MRP4) were synthesized by New England Peptide Inc. (Gardner, MA). The stable isotope C-13 and N-15 were labeled on the C-terminal arginine of each internal peptide standard.

### P-gp-MDR1 Efflux Assay

MDCKII wild-type (MDCK-WT) and MDCKII cells overexpressing P-gp-MDR1 protein (MDCK-MDR1) (licensed from The Netherlands Cancer Institute, the Netherlands) were maintained at 37°C, 95% relative humidity, and 5% CO_2_, and spitted once a week at 80% to 95% confluence. The cells were plated onto 24-well insert plates (HTS-Transwell^®^ inserts, surface area: 0.33 cm^2^, Corning, Bedford, MA) at a density of approximately 500,000 cells/cm^2^ and grown for 4 days. At day 4, the cell inserts were rinsed twice and then incubated for 30–40 min with Hank’s Balanced Salt Solution (HBSS) buffer, pH 7.4. Trans-epithelial electrical resistance (TEER) values were measured in wash buffer and the acceptable value of TEER is >325 (Ω*cm^2^). Compounds were diluted to indicated concentrations in HBSS buffer and applied to the donor chamber for each direction of transport, either apical to basolateral (A→B) or basolateral to apical (B→A). Positive control substrate digoxin (P-gp-MDR1) was included in the studies to confirm P-gp-MDR1 activity. Transporter inhibitors, quinidine (10 µM), and ketoconazole (10 µM) were added to both apical and basolateral chambers. The cell monolayer integrity was tested by adding 200-µl buffer with Lucifer yellow (2 µM) to apical chamber after aspirating all remaining buffer. The acceptance criteria of Lucifer yellow transport was less than 2 × 10^6^ cm/s. The effect of chloroquine on digoxin efflux in Caco-2 monolayers was evaluated at concentrations of 12.5 and 25 µM (Marino et al., 2005). Briefly, Caco-2 cells were grown on 24-well filter insert membrane (Corning, Bedford, MA) at a density of ∼100,000 for approximately 21 days at 37°C, 95% relative humidity, and 5% CO_2_. The culture medium was replaced every 2 days. Bidirectional transport studies were conducted in human epithelial colorectal adenocarcinoma (Caco-2) monolayers with TEER values greater than 400 Ω cm^2^. Plates were incubated at 37°C for 2 h. Samples were collected from both donor and receiver chambers at t = 120 min, and subjected to an liquid chromatography tandem mass spectrometry (LC-MS/MS) analysis. The apparent permeability (*P* *_app_*, cm/sec) of MDCK or Caco-2 cells was determined for both A→B and B→A directions by the following calculation:

$$P_{\mathrm{app}}=\frac{1}{A*C_{D}(0)}*\frac{dM_{r}}{dt}$$

in which *A* = area of filter membrane, *C* *_D_*(*0*) = initial concentration of the test drug, *dM* *_r_* = the amount of transported drug, and *dt* = time elapsed. The efflux ratio (ER) was calculated from (P_app, B→A_)/(P_app, A→B_).

### Tumor Cell Lines

HepG2 (human liver hepatocellular carcinoma), Hep3B2 (human hepatocellular carcinoma), H226 (human lung squamous cell carcinoma), N87 (human gastric carcinoma), and OVCAR3 (human ovary adenocarcinoma) cells were obtained from ATCC (Manassas, VA). KM-H2 cells that overexpress human the CD30 antigen were obtained from Leibniz-Institute DSMZ-German collection of microorganisms and cell cultures (Braunschweig, Germany). Tumor cell lines were cultured in T175-culture flasks with RPMI-1640 medium containing 11% FBS, 1.1% HEPES, and 1.1% penicillin streptomycin.

### Extraction of Membrane Protein and Crude Lysosomal Fraction

The tumor cells were detached from a flask using 0.25% trypsin EDTA, and washed with HBSS buffer prior to membrane protein extraction. The membrane protein fraction was extracted from fresh tumor cell pellets using the Native Membrane Protein Extraction Kit from Calbiochem (San Diego). Briefly, cell pellets were lysed in extraction buffer I containing a proper amount of protease inhibitor cocktail followed by incubation at 4°C for 30 min. The suspension was centrifuged at 16,000 × *g* for 15 min at 4°C. The supernatant containing cytosolic protein was removed and the pellet was re-suspended in extraction buffer II containing the protease inhibitor cocktail mix. After incubating for 30 min at 4°C, the suspension was centrifuged at 16,000 × *g* for 15 min at 4°C. The supernatant containing membrane protein was collected and the protein concentrations of the membrane fractions were determined by a BCA protein assay kit.

Crude lysosomal fraction from each tumor cell line was prepared from fresh cell pellets using the lysosome isolation kit from Sigma-Aldrich. Briefly, cells were washed with ice cold PBS buffer. After washing, the cell pellets were re-suspended in 2.7 volume of 1× extraction buffer from the lysosome isolation kit. The cells were vortexed to achieve an even suspension and homogenized in a 7-ml dounce homogenizer using pestle to achieve 80–85% of breakage. The homogenate was centrifuged at 1,000 × *g* for 10 min. The supernatant was transferred to a new centrifuge tube and centrifuged at 20,000 × *g* for 20 min in micro-centrifuge tubes. The supernatant was discarded and the crude lysosomal fraction pellet was re-suspended in 1× extraction buffer. The fraction obtained was further purified with the aid of calcium chloride, according to the instruction of the isolation kit. Acid phosphatase was tested for the enrichment of lysosomal fraction to ensure the isolation of lysosomal fractions with high yield (> 80%). It is worthwhile noting that the KM-H2 cell line is established from a patient with Hodgkin lymphoma and appears single, round, and mononucleated cells in suspension. The cells are markedly smaller than those of adherent cell lines such as HepG2. As a result, regardless of multiple attempts being made to obtain lysosomal membrane fraction for KM-H2 cells, the protein yield was too low (<1 µg) for the proteomics quantitation; therefore, the expressions of efflux transporters in lysosomal fraction of KM-H2 cells were undetectable in the current investigation.

### Denaturation and Digestion

As described previously (Qiu et al., 2013), an aliquot protein extract containing 40 to 200 µg membrane or crude lysosomal protein (200 µl) was reduced and denatured in 3.6% deoxycholate in 100 mM ammonium bicarbonate buffer with 20 µl of 10 mM dithiothreitol in boiling water for 20 min. The protein was then alkylated with 15 mM iodoacetamide in the dark for 20 min. Trypsin was then added to each sample at a 1:40 of trypsin/protein ratio and the sample was digested at 37°C overnight (>16 h) in a shaking incubator. The digestion was stopped by the addition of an equal volume of water (with 0.2% FA). The reaction mix was centrifuged at 16,000 × *g* and 4°C for 15 min. The supernatant was dried under N2 and reconstituted in 200 µl of 0.1% FA water for LC-MS/MS analysis.

For preparation of calibration curve samples, HSA (2 mg/ml, digested as described above) was used as blank matrix and was spiked with a fixed volume of internal standard mix and peptide standard mix to generate the following target concentrations of 0.16, 0.8, 4, 20, 50, and 100 nM for calibration values.

### LC-MS/MS Analysis of Efflux Transporters BCRP, P-gp-MDR1, MRP2, MRP3, and MRP4

LC-MS/MS analyses were conducted on an API 6500 triple-quadruple mass spectrometer (AB Sciex, Ontario, Canada) coupled to a Shimadzu LC (Nexera) system (Shimadzu, Wood Dale, IL). Chromatography separation was performed on a peptide BEH C18 column (1.7 µ, 2.1 × 100 mm; Waters, Milford, MA) with a gradient elution. Mobile phase included 0.1% FA in water (solvent A) and 0.1% FA in acetonitrile (solvent B). At a flow rate of 0.3 ml/min, the gradient was as follows: 5%B for 1 min, followed by a linear increase to 37%B at 8 min, and reached 90%B at 8.2 min, held 1.8 min at 90%B, and went back to 5%B at 10.2 min. Injection volume for standards and samples was 20 µl. The mass spectrometer was equipped with the electrospray ionization (ESI), Turbolon interface, and operated in the positive mode to monitor the MRM transitions for all peptides and their internal standards (**Table 1**). All chromatograms were evaluated with Analyst 1.6.2 software.

**Table 1:** Quantitative peptides of ABC efflux transporters and their isotope internal standards.

Transporter ID	Peptide	Q1 Mass (Da)	Q3 Mass (Da)	IS Peptide*	Q1 Mass (Da)	Q3 Mass (Da)
BCRP	SSLLDVLAAR	522.9	644.3	SSLLDVLAAR*	527.9	654.3
		522.9	757.3		527.9	767.3
		522.9	870.3		527.9	880.3
P-gp-MDR1	AGAVAEEVLAAIR	635.3	971.4	AGAVAEEVLAAIR*	640.3	981.4
		635.3	900.3		640.3	910.3
		635.3	771.3		640.3	781.3
MRP2	LTIIPQDPILFSGSLR	885.8	665.5	LTIIPQDPILFSGSLR*	889.8	669.5
		885.8	989.4		889.8	997.5
		885.8	441.2		889.8	445.2
MRP3	IDGLNVADIGLHDLR	541.2	697.1	IDGLNVADIGLHDLR*	544.2	701.1
		541.2	754.7		544.2	759.3
		541.2	288.3		544.2	292.4
MRP4	APVLFFDR	482.9	796.5	APVLFFDR*	487.9	806.5
		482.9	697.5		487.9	707.5
		482.9	584.3		487.9	594.3

*Labeled amino acid.

### Cytotoxicity of MMAE to HEPG2, HEP3B2, H226, N87, KM-H2, and OVCAR3

Suspension of each cell line was prepared in culture medium at 3,000 cells/50 µl cell density. Three thousand cells were seeded into each well in 50 µl of culture medium and incubated overnight. Fifty microliters of culture medium containing various amounts of drugs was added to the wells, and incubation was continued for 3 days. Tumor cells were treated with MMAE alone at the concentrations up to 500 nM. The treated cell plates were kept in the incubator for 3 days until analyzed by XTT II assay (Roche Applied Science, Mannheim, Germany) performed in 96-well plates. Fifty microliters of yellow XTT labeling mixture was added to each well. After incubation for 16 h, the resulting orange formazan was spectrophotometrically quantified at 492 nm using a Microplate Reader (Molecular Devices, Sunnyvale, CA). The results were expressed as mean ± SD. Percentage of growth was calculated relative to control (untreated cells) after 3 days of culture with control taken as 100%.

### Cytotoxicity of Brentuximab Vedotin to KM-H2 and HEPG2 Cells

Suspension of KM-H2 cells was prepared in culture medium at 15,000 cells/50 µl cell density; 15,000 KM-H2 cells or 3,000 HEPG2 cells were seeded into each well in 50 µl of culture medium and incubated overnight. Fifty microliters of culture medium containing various amounts of drugs was added to the wells, and incubation was continued for 3 days.

HEPG2 and KM-H2 cells were treated with brentuximab vedotin alone at the concentrations of 0, 0.0005, 0.0025, 0.01, 0.04, 0.165, 0.65, 2.6, 10.4, 41.65, and 166.5 nM, or combined with 2 µM of elacridar, or with 25 µM of chloroquine, or with both 2 µM of elacridar and 25 µM of chloroquine. The treated cell plates were kept in the incubator for 3 days until analyzed by XTT II assay as described above. Statistical differences between two groups were determined by an unpaired t-test in GraphPad Prism (v5.0) (GraphPad Software, Inc.; San Diego, CA). A p-value of less than 0.05 was considered to be statistically significant.

## Results

### P-gp-MDR1 Mediated MMAE Efflux

**Figure 1:**
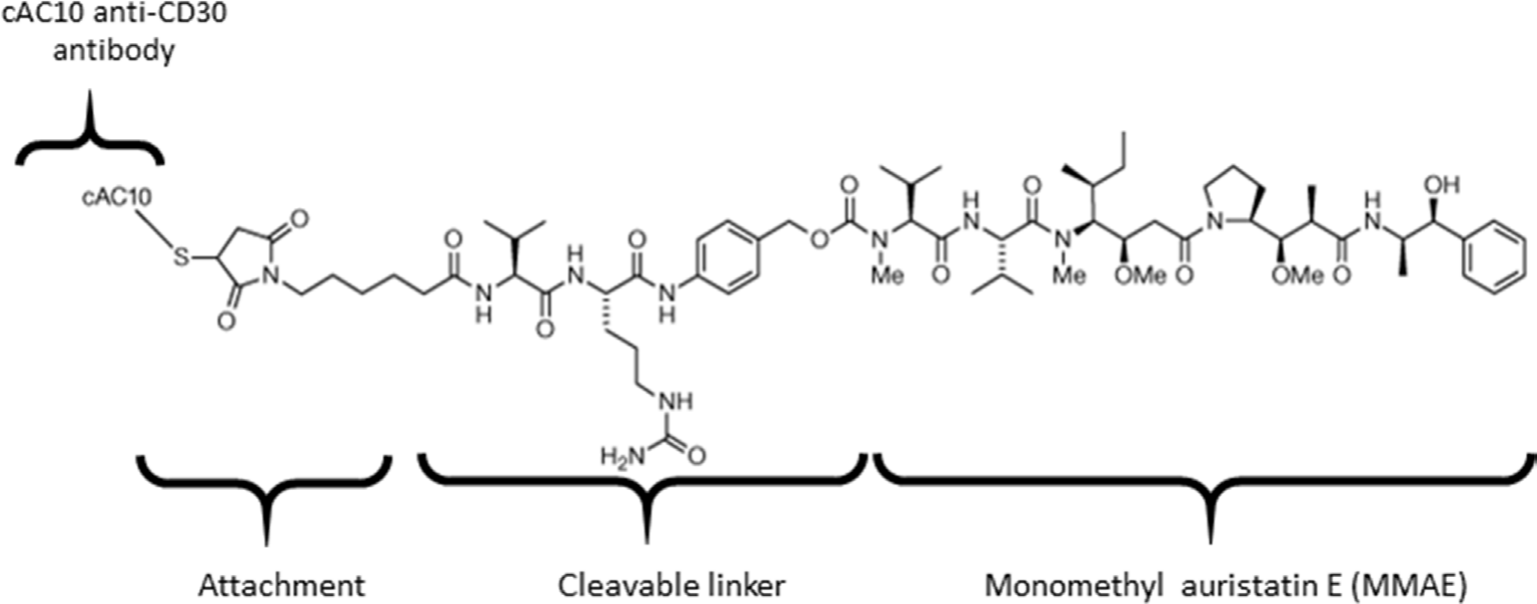
Chemical structure of brentuximab vedotin.

**Figure 2:**
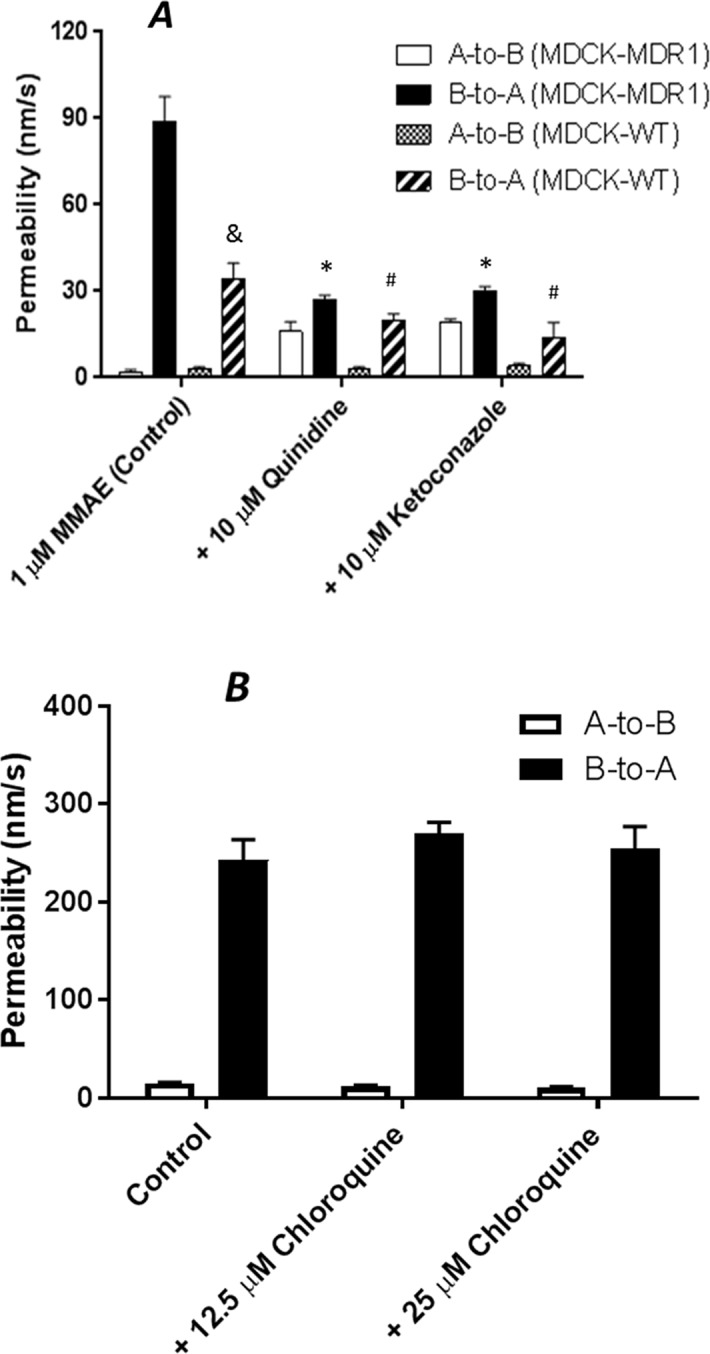
Transepithelial transport of 1 µM MMAE across MDCK-WT and MDCK-MDR1 cell monolayer **(A)**, and 10 µM digoxin across Caco-2 monolayers **(B)**. At 0 min, the test compound with and without a P-gp-MDR1 inhibitor quinidine or ketoconazole (10 µM) for MMAE transport, or chloroquine (12.5 or 25 µM) for digoxin transport was applied into one compartment (either apical or basolateral chamber) and incubated at 37°C. At 120 min, the amount of the compound translocated to the opposite compartment was measured by LC-MS/MS. Transport permeability from the basolateral to the apical compartment (B-to-A) and from the apical to basolateral compartment (A-to-B) were plotted. Data are represented as mean ± SD (n = 3). &: P < 0.05, B-to-A permeability in MDCK-WT as compared to MDCK-MDR1 cells; *: P < 0.05, B-to-A permeability in MDCK-MDR1 with inhibitors as compared to MDCK-MDR1 cells; #: P < 0.05, B-to-A permeability in MDCK-WT with inhibitors as compared to MDCK-WT cells.

Brentuximab vedotin is an ADC that consists of anti-CD30 chimeric antibody attached cytotoxic antimitotic agent MMAE through a cathepsin cleavable linker (**Figure 1**). The antibody of brentuximab vedotin recognizes a specific tumor antigen CD30 and delivers the cytotoxic reagent MMAE to the targeted tumor cells. Once internalized into tumor cells, brentuximab vedotin is hydrolyzed in lysosomes and releases MMAE from lysosomes to inhibit cell division through blocking the polymerization of tubulin. To investigate if efflux transporters are involved in intracellular disposition of MMAE, P-gp-MDR1 mediated efflux of MMAE was firstly determined in MDCK-WT and MDCK-MDR1 cells. As shown in **Figure 2A**, MMAE showed significantly polarized transport in both MDCK-WT and MDCK-MDR1 cells with ERs at 13.6 and 44.5, respectively, resulting ratio of ratios of 3.3. The P-gp-MDR1 mediated efflux was completely eliminated (efflux < 1.5) by known P-gp-MDR1 inhibitors quinidine or ketoconazole at 10 µM each, suggesting that MMAE is a P-gp-MDR1 substrate.

Chloroquine is a lysosomal lumen alkalizer and a lysosomal autophagy inhibitor that impairs lysosomal functions. To determine if chloroquine is a P-gp-MDR1 modulator, P-gp-MDR1-mediated digoxin efflux in Caco-2 cells was co-incubated with chloroquine at concentrations of 12.5 and 25 µM. As shown in **Figure 2B**, P-gp-MDR1-mediated digoxin efflux in Caco-2 cells was not altered by chloroquine, suggesting that chloroquine is not a P-gp-MDR1 inhibitor under the concentrations tested.

### Transporter Expression in Total Membrane and Lysosomal Fraction Extracted from Tumor Cell Lines and the Cytotoxicity of MMAE

Quantitative expressions of efflux transporters were determined in total membrane fractions extracted from HepG2, Hep3B2, H226, N87, OVCAR3, and KM-H2 cells (**Table 2**). Five efflux transporters known to be involved in multidrug resistance including BCRP, MDR1, MRP2, MRP3, and MRP4 were quantified using quantitative proteomics LC-MS/MS method reported previously (Qiu et al., 2016). As showed in **Table 2**, the expressions of ABC efflux transporters varied among the tumor cell lines tested. P-gp-MDR1, MRP2, and MRP4 were highly expressed in HepG2 and Hep3B2 cells. H226 cells highly expressed BCRP and MRP4, while only MRP4 was detected in N87 cells. All efflux transporters except for MRP2 were found in KM-H2 cells. In contrast, the expressions of the efflux transporters in OCAR3 cells were low at under the detection limits.

**Table 2:** Transporter expression in total membrane fractions extracted from tumor cell lines.

Cell lines	Transporter expression in total membrane fraction [fmole/μg protein (SD), n = 3]				
	BCRP	P-gp-MDR1	MRP2	MRP3	MRP4
HEPG2	<LLQ	0.59 (0.06)	0.58 (0.24)	<LLQ	0.22 (0.14)
HEP3B2	<LLQ	0.42 (0.18)	0.17 (0.07)	<LLQ	0.23 (0.09)
H226	0.56 (0.34)	<LLQ	<LLQ	<LLQ	0.54 (0.33)
OVCAR3	<LLQ	<LLQ	<LLQ	<LLQ	<LLQ
N87	<LLQ	<LLQ	ND	<LLQ	0.36(0.0)
KM-H2	0.59 (0.15)	0.12 (0.03)	ND	0.35 (0.21)	0.45 (0.23)

**Figure 3:**
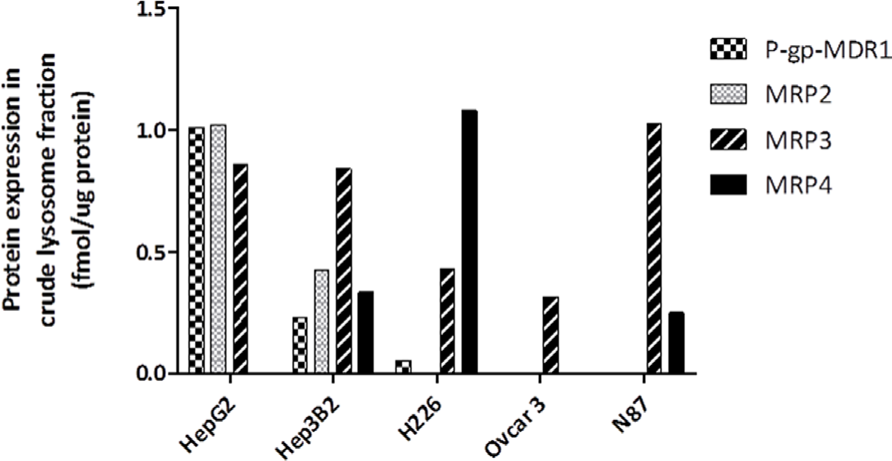
Expression of ABC transporter proteins in the crude lysosomal fraction isolated from HepG2, Hep3B2, H226, OVCAR3, and N87 cells. The data represented the single LC-MS/MS injection for the lysosomal samples extracted from the same batch cells. Although multiple attempts were conducted for KM-H2 cells, the yield of lysosomal fraction for KM-H2 cells was too low, and therefore the lysosomal expression of P-gp-MDR1 in KM-H2 was not included. The variation (CV%) between batch preparation was generally <30%.

The expressions of efflux transporters were also determined in crude lysosomal fraction extracted from the tumor cells (**Figure 3**). In agreement with the P-gp-MDR1 expression in the total membrane fraction, P-gp-MDR1 level was high in crude lysosomal fraction from HepG2 cells. BCRP in the crude lysosomal fraction was not detectable in all tumor cells tested, despite that BCRP expression was found to be high in total membrane fractions of H226 cells. MRP4 expression was found to be high in crude lysosomal fraction of H226 cells.

**Figure 4:**
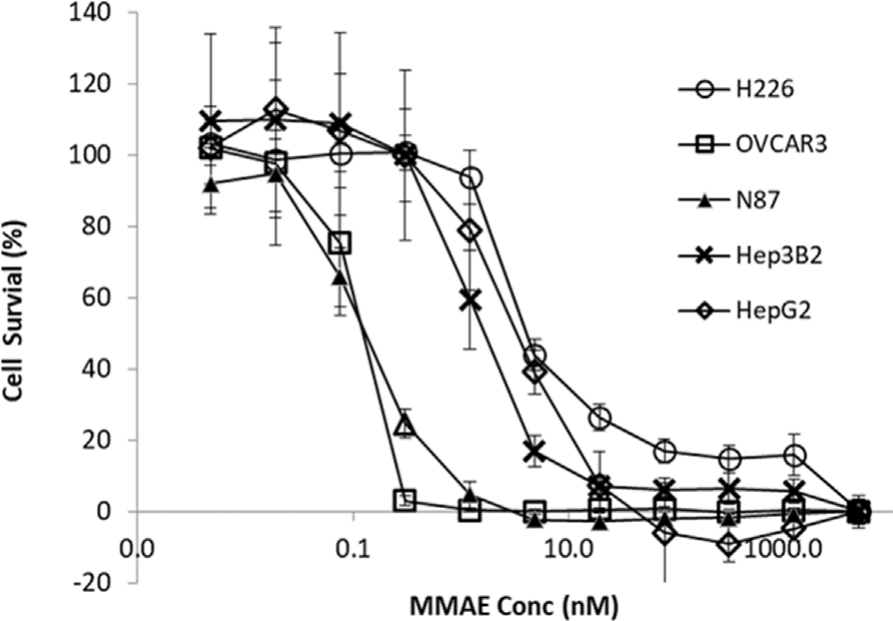
Cytotoxic effects of MMAE on various tumor cell lines. The HepG2, Hep3B2, H226, OVCAR3, KM-H2, and N87 cells were cultured for 3 days at 37°C with MMAE at designated concentrations and then assessed by XTT method. Each point represents the mean value of eight individual assays with SD.

Cytotoxicity of MMAE was determined among the tumor cell lines. As shown in **Figure 4**, compared to N87 and OVCAR3 cells, the cytotoxic effects of MMAE on HepG2, Hep3B2, and H226 appeared to be less sensitive.

### The Impact of P-gp-MDR1 Inhibition or Lysosomal Function on Cytotoxicity Induced by MMAE and Brentuximab Vedotin

Concentration-dependent cytotoxicity of MMAE was determined in the tumor lines. As showed in **Figure 4**, dose-dependent cytotoxicity of MMAE was observed in all tumor cell lines. The EC_50_ (cell killing effects) of MMAE were about three- to fourfold higher in HepG2, Hep3B2, and H226 cells than that in N87 or OVCAR3 cells. As a result, N87 and OVCAR3 cells that do not highly express P-gp-MDR1 appeared to be more sensitive to MMAE than HepG2, Hep3B2, and H226 cells. Elacridar, a potent P-gp-MDR1 inhibitor, potentiated MMAE cytotoxic effects for about 4- to 22-fold in HepG2, Hep3B2, KM-H2, and H226 cells, but the effects on N87 and Ovcar3 cells were generally less than twofold (**Table 3**). On the other hand, chloroquine (25 µM), a lysosomal lumen alkalizer and lysosomal autophagy inhibitor, also enhanced the cytotoxic effects of MMAE on HepG2, Hep3B2, KM-H2, and H226 cells for about 3- to 16-fold, while the EC_50_ of MMAE cytotoxic effects on N87 and OVCAR3 shifted 1.2- and 1.5-fold, respectively (**Table 3**).

**Table 3:** Impact of elacridar or chloroquine on cytotoxic effects of MMAE.

Cell type	Treatment	EC_50_ fold shifted as compared to control
HepG2	+ 25 µM chloroquine	16
	+ 2 µM elacridar	21.9
H226	+ 25 µM chloroquine	3.8
	+ 2 µM elacridar	9.4
Hep3B2	+ 25 µM chloroquine	2.9
	+ 2 µM elacridar	11.8
KM-H2	+ 25 µM chloroquine	2.9
	+ 2 µM elacridar	4.2
N87	+ 25 µM chloroquine	1.2
	+ 2 µM elacridar	2
OVCAR3	+ 25 µM chloroquine	1.5
	+ 2 µM elacridar	1.7

**Figure 5:**
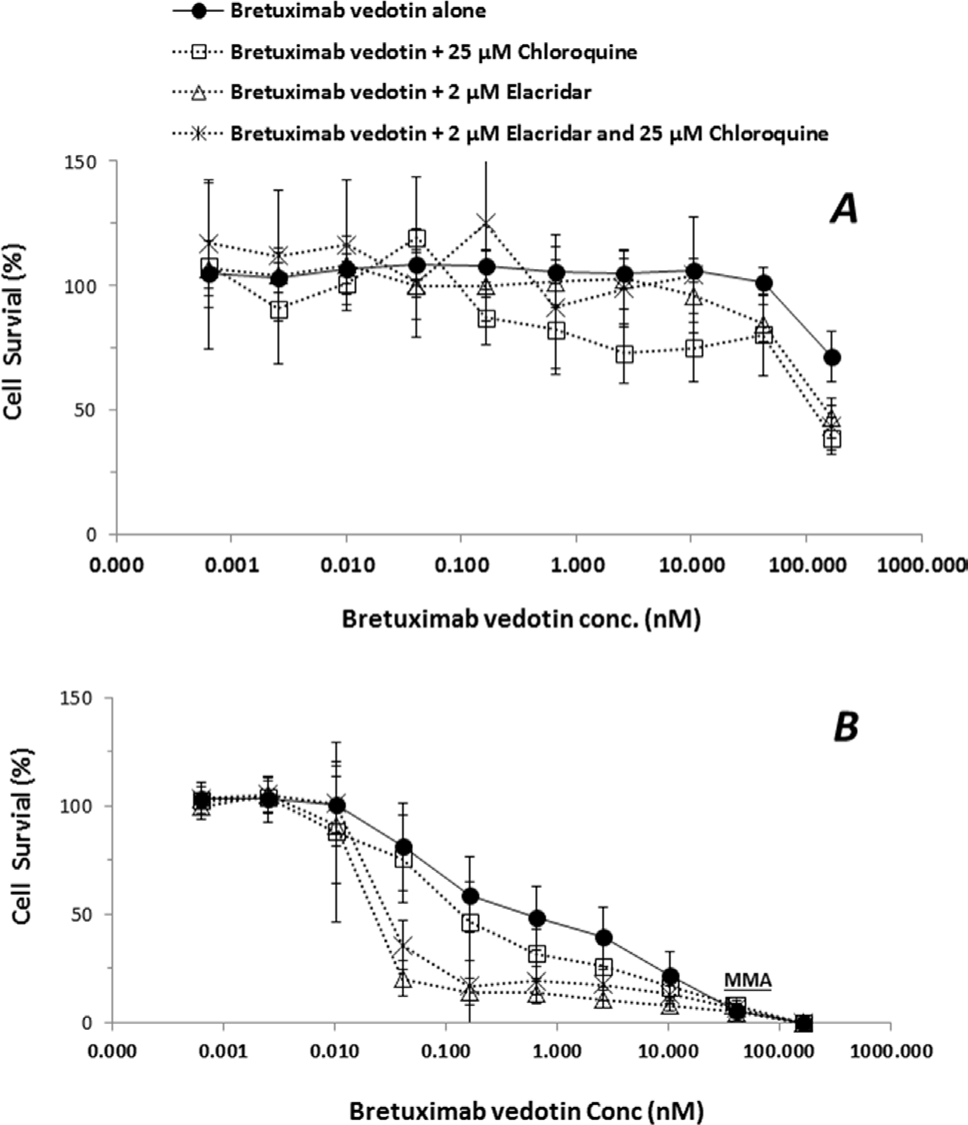
Effects of elacridar (2 µM), chloroquine (25 µM), or the combination (2 µM elacridar plus 25 µM chloroquine) on cytotoxicity of brentuximab vedotin. HepG2 and KM-H2 cells were cultured for 3 days with brentuximab vedotin at designated concentrations in the absence or presence of 2 µM elacridar, 25 µM chloroquine, or the combination. The cell survival was assessed by cytotoxicity kits (XTT method). Each point represents the mean value of eight individual assays with SD. **(A)**, HepG2 cell; **(B)**, KM-H2 cells.

In order to elucidate if the efflux transporter expressions are associated with an ADC payload release from lysosome to cytoplasm, the ADC-induced cytotoxicity was further conducted in KM-H2 cells. HepG2 cells that do not express CD30 antigen were used as negative controls of MMAE delivered by brentuximab vedotin. While cytotoxic effects of brentuximab vedotin were not observed in HepG2 cells with the concentrations up to 200 nM due to lack of CD30 expression, brentuximab vedotin-inducted cytotoxicity in KM-H2 cells appeared to be dose-dependent (**Figure 5**). The cell-killing effects were potentiated by the co-incubation with elacridar (2 µM) or chloroquine (25 µM), or in the combination (**Table 4**). The EC_50_ shift was 2.8-, 21.4-, and 15.6-fold in the presence of elacridar (2 µM) or chloroquine (25 µM), or in combination, respectively (**Table 4**).

**Table 4:** Impact of elacridar and/or chloroquine on cytotoxic effects of brentuximab vedotin.

Cell type	Treatment	EC_50_ (nM)	EC_50_ fold-shifted
HepG2	Brentuximab vedotin alone	ND	
	+ 25 µM chloroquine	ND	
	+ 2 µM elacridar	ND	
	+ 25 µM chloroquine and 2 µM elacridar	ND	
KM-H2	Brentuximab vedotin alone	0.50 ± 0.17	
	+ 25 µM chloroquine	0.18 ± 0.05*	2.8
	+ 2 µM elacridar	0.024 ± 0.05*	21.4
	+ 25 µM chloroquine and 2 µM elacridar	0.032 ± 0.008*	15.6

ND, not determined. *, P < 0.05 as compared to brentuximad vedotin alone.

## Discussion

It has been established that membrane transporters facilitate the transport of endogenous and exogenous molecules across cell membranes, and play important roles in the absorption, distribution, and excretion of many drugs. The MDR is a major limiting factor of chemotherapeutic drug treatment. The most characterized mechanism of MDRs is the up-regulation of ABC efflux transporters that actively transport chemotherapeutic drugs out of cells, resulting in cytoprotection. Remarkably, P-gp-MDR1 transports a wide range of chemotherapeutic drugs, and is the most studied ABC transporter protein involved in the MDR (Kartner and Ling, 1989). During endocytosis, P-gp-MDR1 initially expressed at the plasma membrane is endocytosed into the lysosomes, resulting in localization on the lysosomal membrane facing inward into the lysosome compartment. Lysosomes are acidic intracellular organelles and contain an assortment of enzymes that have catalytic activities at acidic pH and are involved in the degradation and recycling of cellular proteins. Lysosomal membrane contains many membrane proteins that transport substances into and out of the lysosomal lumen, and maintain the lysosomal acidic environment. For example, the lysosomal amino acid transporter 1 is a lysosomal transporter involving the transport of lysine and arginine for cellular amino acid homeostasis (Liu et al., 2012).

ADCs have become a promising class of drugs fighting cancers owing to their ability to specifically recognize tumor specific surface antigens and deliver potent cytotoxic agents to cancer cells. ADCs bound to the surface antigens are internalized following the processes of lysosomal formation where they are catalyzed to release toxic payloads. It is thought that the released cytotoxic payloads freely diffuse across lysosomal membrane to reach a sufficient concentration to kill cancer cells. For example, brentuximab vedotin, the first marketed ADC drug, incorporates a peptide cleavable linker conjugated to MMAE (**Figure 1**), selectively binds to tumor cells expressing CD30 antigen for the treatment of relapsed or refractory Hodgkin lymphoma and systemic anaplastic large cell lymphoma. In the lysosomes, the amide bond between the citrulline residue and the p-aminobenzyl carbamate portion of the linker is cleaved to release MMAE by lysosomal proteases. Recently Hamblett et al. (2015) demonstrate that SLC46A3, a folate transporter, is required to transport the ADC payload maytansine from the lysosome to the cytoplasm. The impact of lysosomal proteins on MMAE transport across the lysosomal membrane has not been fully characterized. Specifically, the role of P-gp-MDR1 protein expressed on the lysosomal membrane on the acquired MDRs remains uninvestigated.

MMAE is a P-gp-MDR1 substrate. While it is well known that ABC transporters expressed on plasma membrane facilitate the efflux of many anticancer drugs from tumor cells, resulting in MDR, the inward facing lysosomal P-gp-MDR1 therefore can sequester its substrates in the lysosomes and prevent the cytotoxic payloads from reaching their intracellular targets such as nucleus and microtubules (Yamagishi et al., 2013). It is known that P-gp-MDR1 on the lysosomal membrane is able to transport cytotoxic agents such as doxorubicin into the organelle and results in MDRs (Seebacher et al., 2016a; Seebacher et al., 2016b). However, the role of P-gp-MDR1 on the lysosomal membrane on ADC payload release from lysosomes to cytoplasm remains uninvestigated. To elucidate the impact of ABC transporters on the ADC payload transport across the lysosomal membrane, we firstly quantify the transporter expressions and investigate the cytotoxic effects of MMAE in various tumor cell lines. Overexpression of P-gp-MDR1 was determined in HepG2 and Hep3B2 cells, both in plasma membrane and lysosomal membrane fraction. Accordingly, the cells were less sensitive to the cytotoxic agent, MMAE (**Figure 3**). The cytotoxic effect of MMAE was potentiated by the treatment of elacridar, a potent inhibitor of P-gp-MDR1. Since elacridar can inhibit P-gp-MDR1 expressed on both the lysosomal and plasma membrane, reduced P-gp-MDR1 activity on the lysosomal membrane could increase the MMAE transport across the lysosomal membrane to cytoplasm. On the other hand, inhibition of plasma P-gp-MDR1 activity could also reduce the extrusion of MMAE from the tumor cells, thereby increasing intracellular exposure leading to greater cytotoxic effects. As a result, P-gp-MDR1 inhibition in cell-based experiments would not be able to differentiate if the potentiated MMAE effects were attributed mainly by the increased MMAE release from the lysosomes due to the inhibition of lysosomal P-gp-MDR1, or blocking MMAE efflux from the cells, or both. Therefore, lysosomal modulators that can impact lysosomal activity, but not P-gp-MDR1 function, can delineate the role of efflux transporters on lysosomal membranes toward cytotoxic effect of payload like MMAE. Chloroquine is a lysosomotropic amine, and can partially reverse the drug resistance of KB carcinoma cells to many cytotoxic chemotherapeutic reagents such as adriamycin, daunomycin, and vincristine (Shiraishi et al., 1986). However, the exact reversal mechanism of cytotoxic effects of lysosomotropic reagent remains unknown. In the present investigation, we demonstrated that chloroquine is not an inhibitor for P-gp-MDR1 when tested at 25 µM (**Figure 2** **B**). Thus, the reversed cytotoxic effects of chloroquine are unlikely the results of inhibition of P-gp-MDR1 activity. It has been reported that chloroquine is protonated in lysosomes, resulting in increased lysosomal pH and inactivation of lysosomal enzymes (Wang et al., 2015). Since P-gp-MDR1 function is also pH-dependent (Varma and Panchagnula, 2005), and the increased lysosomal pH can reduce P-gp-MDR1 activity on lysosomal membrane, it leads to the increased cytotoxic effects of MMAE on the tumor cells that highly express P-gp-MDR1. It is worth noting that H226 cells overexpressed BCRP and MRP4, but not P-gp-MDR1. However, the cells also showed resistance to MMAE and, similar to HepG2 and Hep2B3 cells, the cytotoxic effects were reversed by elacridar and chloroquine. Since elacridar is also a potent inhibitor for BCRP, investigation of its role or other potential mechanisms on MMAE resistance are warranted.

To further elucidate the effects of lysosomal P-gp-MDR1 on MMAE sequestration directly released from an ADC drug, HepG2 and KM-H2 cells were treated with brentuximab vedotin. As expected, since HepG2 cells do not express the CD30 antigen, the treatment of brentuximab vedotin had no effects on HepG2 survival (**Figure 5**). In contrast, concentration-dependent cell-killing effects of brentuximab vedotin were observed in KM-H2 cells. In addition, the cell-killing effects of the ADC were potentiated about 2.8-fold by 25 µM chloroquine in KM-H2 cells. Co-incubation with elacridar or the combination of elacridar and chloroquine could further increase susceptibility of KM-H2 cells to brentuximab vedotin (**Figure 5** and **Table 4**). As shown in **Table 2** and **Figure 3**, P-gp-MDR1 but not BCRP expressions in lysosomal membrane from HepG2 and Hep3B2 cells were correlated with the expressions in plasma membrane preparations. Although P-gp-MDR1 expression in lysosomal membrane of KH2 cells was not able to obtain due to technique issues in sample preparations, the assumption on lysosomal P-gp-MDR1 expression in KH2 cells was valid based on the P-gp-MDR1 expression data detected in the plasma membrane fraction. These results suggest that the inhibition of P-gp-MDR1 activities at plasma membrane and/or lysosomal membrane can enhance the accessibility of the microtubule-binding agent to its target.

In conclusion, tumor cells that highly express P-gp-MDR1 on the plasma and/or lysosomal membranes show resistant to the cytotoxic ADC payload, MMAE. The flux mediated by P-gp-MDR1 on plasma and/or lysosomal membrane implicates the attenuated cancer-killing activities of MMAE and brentuximab vedotin. P-gp-MDR1 located on the lysosomal membrane is an in-ward facing transporter and can sequester MMAE that is released from the ADC brentuximab vedotin in the lysosomes, which prevents it from reaching to its cytosolic targets. The results revealed that, to avoid the efflux transporter mediated MDR, strategies to design ADC drugs for specifically delivering cytotoxic reagents to targeted cancer cells through the selective binding of mAbs may no longer be viable approaches. Understanding the subcellular expression of efflux transporters in the intended targeted cancers and assessing transporter substrate potential for ADC payloads become critical for driving the success of ADCs discovery and development.

## Data Availability

The raw data supporting the conclusions of this manuscript will be made available by the authors, without undue reservation, to any qualified researcher.

## Author Contributions

PL-K, HS, and YL participated in research design. PL-K, AM and HS conducted the experiments and performed data analysis. PL-K, HS, WH, AM, YL, and RI contributed to the writing of the manuscript.

## Conflict of Interest Statement

The authors were all employees of Bristol-Myers Squibb during this research.
